# Perceptions of mental health services among informal caregivers in Sardinia, Italy, during the post-pandemic crisis of the national health system: a comparative study between 2024 and 2025

**DOI:** 10.3389/fpsyt.2026.1738728

**Published:** 2026-05-29

**Authors:** Noemi Mereu, Gabriele Sole, Massimo Tusconi, Michela Atzeni, Giulia Cossu, Viviane Kovess-Masfety, Mauro Giovanni Carta

**Affiliations:** 1 Doctoral School in Capacities Building for Global Health, Department of Medical Sciences and Public Health, University of Cagliari, Cagliari, Italy; 2 Center for Liaison Psychiatry and Psychosomatics, University Hospital Of Cagliari, Cagliari, Italy; 3 Department of Medical Sciences and Public Health, University of Cagliari, Cagliari, Italy; 4 Université Paris Cité, Laboratoire de Psychopathologie et Processus de Santé (LPPS), Paris, France

**Keywords:** community psychiatry, human rights, informal caregivers, Italy, mental health services, organizational well-being, WWRR questionnaire

## Abstract

**Background:**

Italy’s community-based mental health model, grounded in Law 180, has long been regarded as a global example of rights-oriented psychiatric reform. However, recent years have witnessed a crisis in the national health system, marked by chronic and progressively accumulating underfunding, workforce shortages, and organizational strain, rather than a single acute disruption. This s/tudy compares two samples of informal caregivers of individuals receiving mental health care in Sardinia (2024 vs. 2025) to explore differences in their perceptions of service quality, organizational well-being, and respect for human rights.

**Methods:**

A cross-sectional design was used to compare independent caregiver samples (2024: *n* = 100; 2025: *n* = 74). Participants completed the *Well-Being at Work and Respect for Human Rights Questionnaire (WWRR)*, which assesses satisfaction with care, perceptions of organizational quality, and respect for users’ and staff’s rights. Data were analyzed with ANOVA and chi-square tests.

**Results:**

Caregivers in 2025 reported significantly higher satisfaction than those in 2024 regarding the quality of services (*p* = 0.007), organizational aspects (*p* = 0.007), and respect for the human rights of both users (*p* < 0.001) and staff (*p* = 0.037). No differences emerged in perceived user satisfaction or resource adequacy, with both cohorts expressing persistent concern about staffing and infrastructure. The perceived need for additional nurses, doctors, and support staff increased in 2025, which may indicate growing awareness of workforce fragility.

**Conclusions:**

These differences in satisfaction should be interpreted within a context of prolonged systemic strain, which may foster adaptive or resilience-based perceptions among informal caregivers. Despite ongoing structural difficulties, informal caregivers maintain high confidence in Italy’s community mental health services and perceive them as respectful of human rights. However, their increasing concern about resource shortages highlights the urgent need for investment to preserve the ethical and organizational strengths of the Italian model.

## 1 Introduction

Italy’s Law 180 (Basaglia Law) initiated one of the world’s most radical psychiatric reforms by closing psychiatric hospitals and establishing community-based mental health services (1–3). This rights-centered model emphasizes social inclusion, ethical care, and the respect of the dignity of persons with psychosocial disabilities (4–7).

Over the past decade, however, Italy’s National Health Service has experienced mounting challenges, reduced public spending, workforce shortages, and bureaucratic inefficiencies, that have raised questions about the sustainability of community mental health care (8–11). The same crisis has affected social services and the entire welfare system (12–15), which has a great influence on community mental health work (16–19).

The post-pandemic period has therefore seen widespread dissatisfaction by users and the entire community against a healthcare delivery system, perceived as increasingly inefficient and incapable of responding to needs (20–24). This discontent is increasingly being directed at those who interface with citizens, namely healthcare professionals themselves, who are often seen not as victims of the lack of resources, but as culprits of the dysfunctions (25–29). This is happening to healthcare workers who are already in precarious conditions, due to the trauma suffered during the recent pandemic, often with long-term consequences, (30–33).

This erosion of confidence in institutions has extended to mental health, reshaping public attitudes toward psychiatric reform and human rights (34–36). In the climate of threat generated by general uncertainty, social tension, and economic crisis, even the “mad” is seen as potentially dangerous, and all of this is reflected in a part of the public with the desire to reopen asylums and the view of mental illness and treatments, which has sparked a subsequent academic (37–39) and media debate (40, 41).

The perception of climate and respect for rights in healthcare services has been investigated in a series of studies conducted in Sardinia and other parts of the world using the Well-Being at Work and Respect for Human Rights Questionnaire (WWRR) (42–47). This instrument is inspired by the WHO QualityRights project (48–52) and the UN Convention on the Rights of Persons with Disabilities. The underlying concept is that the quality-of-care services is closely linked to respect for the rights of users and providers within those services (42).

Studies conducted in Sardinia using this instrument have revealed surprisingly given the described picture, that informal caregivers, users, and professionals generally express high satisfaction and perceive strong respect for human rights within mental health services, despite recurring concerns about insufficient resources (44, 53).

However, the cited growing financial and political instability affecting the funding of the Italian healthcare system, combined with a climate of unrest in recent months that has seen politicians and social actors take positions no longer favorable to mental health care outside of psychiatric hospitals, may have influenced these perceptions.

Against this backdrop of chronic underfunding and workforce shortages, informal caregivers have become an increasingly central component of the Italian mental health system, often assuming a de facto compensatory role in sustaining continuity of care. Despite their crucial contribution, limited evidence is available on how caregivers’ perceptions of service quality, organizational functioning, and respect for human rights may differ between independent cross-sectional samples collected under different healthcare system conditions (e.g., 50). Despite this central role, little is known about how caregivers’ perceptions of service quality, organization, and human rights may reflect differences between independent cross-sectional cohorts observed under conditions of increasing systemic strain. Understanding these dynamics is particularly relevant in Italy, a country that pioneered deinstitutionalization and community-based psychiatry. Importantly, the present study is situated within a phase of chronic and cumulative strain affecting the Italian National Health Service, rather than a single acute crisis. This prolonged condition of underfunding and workforce shortages may shape caregivers’ perceptions in complex ways, potentially fostering adaptive responses and recalibration of expectations over time (54). In this context, the present study provides a comparative analysis of two independent cross-sectional samples of informal caregivers surveyed in 2024 and 2025. The aim is to explore whether and how caregivers’ perceptions of service quality, organizational well-being, and respect for human rights may differ under conditions of sustained systemic pressure. The present study investigates differences between two independent samples of informal caregivers of individuals with psychosocial disabilities surveyed in 2024 and 2025.

## 2 Methods

### 2.1 Study design and participants

A cross-sectional design was used to compare two independent cohorts of informal caregivers of individuals receiving mental health care in Sardinia, Italy. Caregivers were relatives or close friends of service users attending the same mental health centers that participated in both data collections. The 2024 survey, previously described by Atzeni et al. (44, 55, involved caregivers recruited from four territorial mental health centers in Southern Sardinia, Sanluri, Carbonia, and two facilities in Cagliari (ASARP, Sardinian Association for the Implementation of Psychiatric Reform; and AOU-CA, University Hospital of Cagliari). The study adopted a semi-anonymized data collection procedure. Caregivers were identifiable to the recruiting clinicians at the time of participation, which allowed the research team to ensure that each individual could participate only once, however, no directly identifying information was retained in the analytical dataset. Recruitment occurred during routine service access and participation was limited to one caregiver per contact episode. Participants in both waves were recruited through the collaborating mental health centers during routine service activities. Informal caregivers were approached in waiting areas or during scheduled visits and were invited to participate on a voluntary basis. Recruitment was facilitated by healthcare staff who informed potential participants about the study, while ensuring that participation was entirely independent and without any influence on the care provided. To enhance comparability between the two samples, the same recruitment settings, procedures, and inclusion criteria were maintained across the 2024 and 2025 data collections. However, given the use of convenience sampling, it cannot be excluded that unmeasured differences in caregiver characteristics may have influenced participation.

Participants were recruited among informal caregivers attending the participating mental health services on a voluntary basis. Given the semi-anonymized recruitment procedure and the restriction of one participation per caregiver, responses were treated analytically as independent observations, consistent with the study design. Nevertheless, because the dataset did not include a formal caregiver–service user linkage variable, the possibility that more than one caregiver referred to the same service user cannot be completely excluded. This aspect should be considered when interpreting the results. This approach reflects a real-world sampling strategy aimed at capturing caregivers’ perceptions within routine clinical contexts, albeit with inherent limitations in representativeness.

The 2025 survey was conducted in three of these centers, using identical recruitment procedures and inclusion criteria to ensure comparability. Participation was voluntary, and all respondents provided written informed consent.

- 2024 convenience sample: 100 caregivers recruited from four territorial mental health centers (44, 55).
- 2025 convenience sample: 74 caregivers recruited from three of the same centers using identical inclusion criteria and survey methods.

Inclusion criteria were: (a) being an adult (≥18 years) informal caregiver. Sex was recorded according to a binary classification (male/female) as part of routine data collection procedures within participating services. No specific measures were implemented to capture gender identity beyond this classification, and therefore gender-diverse individuals (e.g., non-binary or transgender participants) may not be adequately represented; (b) ability to read and understand Italian; and (c) voluntary participation with written informed consent. Exclusion criteria were: (a) being a paid or professional caregiver; (b) cognitive or linguistic difficulties preventing questionnaire completion; and (c) absence of a formal link to the centers involved in the study.

It is important to note that the two samples (2024 and 2025) are two cross-sectional caregiver samples that were treated as independent samples rather than repeated measures on the same individuals. Therefore, the comparison reflects differences between two independent samples rather than longitudinal change within the same individuals. This design allows for the exploration of differences in perceived service quality and human rights within a real-world context, although causal inferences cannot be drawn.

The study was conducted in accordance with internationally recognized ethical principles for research involving human participants, including those outlined in the Declaration of Helsinki. The 2025 survey was approved by the Regional Ethics Committee of Sardinia (Protocol 25569, 18 September 2025).

### 2.2 Instruments

The Well-Being at Work and Respect for Human Rights Questionnaire (WWRR), previously validated in multiple studies (42, 43, 47, 56), was administered in its caregiver version (55). The instrument comprises six core items rated on a six-point Likert scale (1 = not satisfied at all to 6 = completely satisfied), assessing key aspects of well-being and perceived respect for human rights in the workplace. The observed internal consistency (Cronbach’s α = 0.691) falls within the acceptable range for exploratory research, particularly for instruments assessing complex and multidimensional constructs such as well-being and perceived respect for human rights. The WWRR captures conceptually related but distinct domains, which may contribute to a moderate level of internal consistency. Nevertheless, its prior validation and use in international studies support its adequacy for assessing caregivers’ perceptions in the present context.

1. Satisfaction with the care received by one’s relative/friend.
2. Perceived satisfaction of service users.
3. Satisfaction with organizational aspects of services.
4. Perceived respect for users’ human rights.
5. Perceived respect for staff’s human rights.
6. Evaluation of available resources (reverse-scored).

Item 7 is open-ended, asking which professional roles should be increased (e.g., doctors, nurses, psychologists).

Demographic variables collected in the study included sex, age, and education level. Sex was recorded according to a binary classification (male/female) as part of routine data collection procedures within participating services. Age was recorded in years. Education level was assessed through the variable “at least graduated,” which refers to participants who had completed university-level education and obtained a university degree according to the Italian educational system.

### 2.3 Statistical analysis

Descriptive and inferential statistical analyzes were conducted to compare the responses provided by caregivers of people cared for in mental health services in 2025 with those from the 2024 sample previously published by Atzeni et al. (44).

Given the exploratory nature of the study, the analytical strategy was primarily descriptive and comparative. Inferential statistics were used to facilitate the comparison of group-level tendencies between the two cross-sectional samples rather than to test causal hypotheses. The analyzes should therefore be interpreted as descriptive-comparative rather than confirmatory. This approach was considered appropriate for exploring potential differences in caregivers’ perceptions across the two survey waves.

Although the WWRR items are based on Likert-type scales and may deviate from normality due to their ordinal nature, they were treated as approximately continuous variables for the purposes of analysis. This approach is supported by methodological literature indicating that Parametric tests such as ANOVA are commonly applied to Likert-type scales and are generally considered robust to moderate departures from normality in samples of this size. In addition, the use of ANOVA facilitates comparability with previous studies employing the same instrument and allows for straightforward interpretation of mean differences between groups. Nevertheless, the potential impact of non-normal distributions should be considered when interpreting the results.

While the WWRR items may exhibit deviations from normality, the analytical approach adopted in this study was primarily descriptive and comparative in nature. Parametric tests were employed to facilitate the comparison of group-level tendencies between independent samples and should therefore be interpreted as descriptive-comparative indicators rather than confirmatory tests of causal relationships. Accordingly, the results are intended to provide exploratory insight into potential differences between survey waves rather than precise inferential estimates derived from strictly independent observations, and should be interpreted in this context.

Given the independent nature of the samples, the analyzes were designed to detect differences in group-level distributions rather than within-subject differences between samples. In addition to maintaining identical recruitment procedures, we examined the comparability of the two samples across key sociodemographic variables (age, sex, and education), which did not differ significantly. However, other potentially relevant characteristics, such as the clinical severity of the care recipient, duration and intensity of caregiving, and level of functional impairment, were not systematically assessed. These factors may influence caregivers’ perceptions and represent a potential source of residual confounding.

Sex and education level were explored through additional Chi-square analyzes of high WWRR scores. These variables were not included as covariates in the primary ANOVA models due to the exploratory nature of the study, the relatively limited subgroup sizes, and the risk of unstable estimates and overinterpretation in stratified analyzes.

The two samples were treated as independent cross-sectional cohorts. Given the voluntary nature of participation and the time interval between surveys, the likelihood of substantial overlap is considered low.

The analytical strategy assumes independence of observations; however, this assumption cannot be formally verified within the present design. Although the two samples were treated as independent cross-sectional cohorts, the possibility of partial overlap or clustering within the same care context cannot be excluded. This limitation should be considered when interpreting the results.

For items 1–6 of the Well-Being at Work and Respect for Human Rights Questionnaire (WWRR), which are rated on a six-point Likert scale, mean scores and standard deviations were calculated for each year. Comparisons between the two independent samples (2024 and 2025) were performed using one-way analysis of variance (ANOVA) for continuous data and chi-square tests for categorical variables. Accordingly, the statistical analyzes were primarily intended to identify descriptive differences between groups rather than to provide precise inferential estimates. Statistical significance was set at *p* < 0.05.

## 3 Results

### 3.1 Sample characteristics

The 2025 sample (74 participants) included 33.8% men, mean age = 57.4 ± 12.5 years, while the 2024 sample (100 participants) included 21.8% men, mean age = 55.7 ± 14.5 years. Differences in age, sex, and education were not statistically significant (*p* > 0.05). (see **Table 1**).

**Table 1:** Sociodemographic characteristics of two samples of caregivers of individuals supported by mental health services (comparison between Atzeni et al., 2024, and the 2025 sample).

Variable	Data	Caregivers mental health 2025 (N = 74)	Caregivers mental health 2024 (N = 100)	Statistics
Sex (Men)	n (%)	25 (33.8%)	21 (22.0%)	χ² (1 df) = 2.995, *p* = 0.084
Age (years)	M ± SD	57.42 ± 12.53	55.74 ± 14.51	ANOVA (1,172 df): F = 0.639, *p* = 0.425
At least graduated	n (%)	14 (18.9%)	20 (19.8%)	χ² (1 df) = 0.032, *p* = 0.859

N, Number of participants; n, Subsample size; %, Percentage; M, Mean; SD, Standard deviation; χ², Chi-square value; df, Degrees of freedom; F, Fisher’s F statistic (one-way ANOVA); *p*, Probability value (two-tailed significance).

### 3.2 Perceptions of care and human rights (WWRR items 1–6)

Exploratory cross-tabulation analyzes examining high WWRR scores according to sex and educational level showed a significant association only between sex and WWRR1 scores, with female caregivers more frequently reporting high satisfaction with the care received by their relative/friend in the overall sample (p = 0.001). Consistent patterns were also observed when the analyzes were conducted separately within the 2024 and 2025 cohorts. The remaining comparisons were not statistically significant, although trend-level associations emerged for WWRR3 and WWRR4 according to education level. Overall, these additional analyzes did not substantially modify the interpretation of the main findings and support the relative consistency of caregivers’ perceptions across demographic subgroups (**Table 2**).

**Table 2:** Exploratory crosstab analyzes of high WWRR scores according to sex and education level among informal caregivers.

Variable	Male high scores n (%)	Female high scores n (%)	p-value	Non-graduated high scores n (%)	Graduated high scores n (%)	p-value
WWRR1	47 (31.1%)	104 (68.9%)	0.001	125 (83.3%)	25 (16.7%)	0.484
WWRR2	46 (32.9%)	94 (67.1%)	0.092	117 (84.2%)	22 (15.8%)	0.252
WWRR3	47 (33.3%)	94 (66.7%)	0.149	119 (85.0%)	21 (15.0%)	0.078
WWRR4	54 (36.0%)	96 (64.0%)	0.910	120 (80.5%)	29 (19.5%)	0.075
WWRR5	52 (33.8%)	102 (66.2%)	0.106	126 (82.4%)	27 (17.6%)	0.841
WWRR6	24 (42.1%)	33 (57.9%)	0.228	47 (83.9%)	9 (16.1%)	0.742

High scores were defined as scores ≥5 for WWRR items 1–5 and ≥5 for WWRR6 (greater perceived resource inadequacy). Chi-square tests were used to explore associations between WWRR items and sex or education level (“at least graduated”).

Caregivers in the 2025 sample reported higher satisfaction scores compared to those in the 2024 sample on Items 1 (How satisfied are you with the health service in which; P = 0.007); 3 (How satisfied are you with the organizational aspects of the work of the health service in which your relative/friend is cared? P = 0.007); 4 (To what extent do you believe that the human rights of the people cared in the health service in which your relative/friend is cared are respected?, p<0.001); 5 (To what extent do you believe that the human rights of the staff working in the health service in which your relative/friend is cared are respected? (p = 0.037). Items 2 (perceived user satisfaction) and 6 (resource adequacy) showed no significant change. In both years, Item 6 scores reflected persistent dissatisfaction with available resources, indicating continuing structural weaknesses (**Table 3**).

**Table 3:** Answers to items 1–6 of the WWRR in caregivers of individuals supported by mental health services (comparison between Atzeni et al., 2024, and the 2025 sample).

Research sample	Data	Caregivers MH 2025 (74)	Caregivers MH 2024 (100)	ANOVA F (1, 173df)	p-value
WWRR 1 - How satisfied are you with the health service in which your relative/friend is cared	M ± SD	5.54 ± 0.68	5.2 ± 0.9	7.471	0.007
WWRR 2 - How much you believe that the users of the health service in which your relative/friend is cared are satisfied?	M ± SD	5.37 ± 0.94	5.1 ± 1.0	3.261	0.072
WWRR 3 - How satisfied are you with the organizational aspects of the work of the health service in which your relative/friend is cared?	M ± SD	5.54 ± 0.80	5.1 ± 1.2	7.487	0.007
WWRR 4 - To what extent do you believe that the human rights of the people cared in the health service in which your relative/friend is cared are respected?	M ± SD	5.75 ± 0.48	5.2 ± 1.0	19.105	<0.001
WWRR 5 - To what extent do you believe that the human rights of the staff working in the health service in which your relative/friend is cared are respected?	M ± SD	5.54 ± 0.66	5.3 ± 0.8	4.428	0.037
WWRR 6 - How do you evaluate the current state of health service in which your relative/friend is cared? (reverse-scored)	M ± SD	3.31 ± 1.92	3.6 ± 1.5	1.251	0.265

N, Number of participants; M, Mean; SD, Standard deviation; df, Degrees of freedom; F, Fisher’s F statistic (one-way ANOVA); *p*, Probability value (two-tailed significance); WWRR, Wellbeing, Work, and Rights of Relatives questionnaire.

### 3.3 Perceived staffing needs (WWRR item 7)

In 2025, caregivers most frequently indicated nurses (44.6%) as the professionals most urgently needed, compared with 20% in 2024 (p = 0.040). Requests for personal care staff (OSS; 5.4% vs 0%) and for administrative/management staff (5.4% vs 0%) also newly appeared (both Fisher’s exact p = 0.030). Conversely, the proportion indicating psychologists decreased sharply (27.0% in 2025 vs 56.0% in 2024; p < 0.001) (**Table 4**).

**Table 4:** Answers to item 7 of the WWRR in caregivers of individuals supported by mental health services (comparison between Atzeni et al., 2024, and the 2025 sample).

Category	Caregivers MH 2025 (N = 74) n (%)	Caregivers MH 2024 (N = 100) n (%)	Statistics
Medical doctors	26 (35.1%)	48 (48.0%)	χ² (1 df) = 2.685, *p* = 0.101
Nurses	33 (44.6%)	20 (20.0%)	χ² (1 df) = 4.215, *p* = 0.040
OSS – professionals for personal care	4 (5.4%)	0 (0.0%)	Fisher’s Exact test, *p* = 0.030
Psychologists	20 (27.0%)	56 (56.0%)	χ² (1 df) = 13.509, *p* < 0.001
Occupational therapists/ educators/rehabilitation technicians	11 (14.9%)	24 (24.0%)	χ² (1 df) = 2.113, *p* = 0.146
Social workers	6 (8.1%)	17 (17.0%)	χ² (1 df) = 2.847, *p* = 0.092
Administrative/ management staff	4 (5.4%)	0 (0.0%)	Fisher’s Exact test, *p* = 0.030
Security staff	0 (0.0%)	0 (0.0%)	χ² (1 df) = 0, *p* = 1.000
None needs to be incremented	0 (0.0%)	0 (0.0%)	χ² (1 df) = 0, *p* = 1.000
All need to be incremented or multiple types of workers need to be increased	0 (0.0%)	0 (0.0%)	χ² (1 df) = 0, *p* = 1.000

N, Number of participants; n, Subsample size; %, Percentage; χ², Chi-square value; df, Degrees of freedom; *p*, Probability value (two-tailed significance). Multiple responses were allowed. Fisher’s Exact test was applied when expected frequencies were below 5.

An additional exploratory logistic regression analysis confirmed that female sex remained significantly associated with high WWRR1 scores after adjustment for age, education level, and survey year (OR = 5.76, 95% CI 1.95–17.05, p = 0.002).

## 4 Discussion

This comparison between two independent samples suggests that caregivers’ trust in mental health services remains relatively strong despite ongoing systemic pressures. Satisfaction scores for service quality, organizational aspects, and respect for human rights are slightly higher in the 2025 sample compared to the 2024 sample, although the magnitude of these differences is modest, indicating that community mental health services in Sardinia continue to embody the values of dignity, proximity, and inclusion central to the Italian psychiatric reform. Although several differences reached statistical significance, the magnitude of these differences was relatively modest, suggesting that their practical or clinical relevance should be interpreted with caution.

The Italian experience in mental health represents one of the most significant international examples of valuing human and social capital in care processes. Around the Basaglia reform in 1978, Italy developed a model grounded not only in the closure of psychiatric institutions, but above all in the centrality of the person, relationships of proximity, community-based services, and the community itself as a concrete space of inclusion, support, and citizenship. Within this framework, Italian human capital has been expressed through the capacity of professionals, families, caregivers, and local networks to address mental vulnerability not as mere custodial care, but as a shared responsibility, a practice of emancipation, and a recognition of rights. For this reason, Italy has long been regarded as an international reference model for deinstitutionalization and for the development of community-based mental health services, particularly following the Basaglia law, which helped orient European policies and international debate toward more humane, territorial, and participatory approaches (57).

This legacy, however, now appears increasingly at risk. In recent years, the proportion of healthcare expenditure devoted to mental health has declined from 5% in 2009 to below 3% in the post-pandemic period, jeopardizing precisely those community-based services that made the Italian experience exemplary (50, 58). This reduction has not only organizational and financial consequences, but also reflects a broader political and moral crisis concerning the protection of the rights of the most vulnerable individuals. In this context, communities and caregivers take on an even more crucial role: they are not merely a subsidiary resource, but a structural component of the Italian model, as they embody emotional continuity, social memory, everyday proximity, and the capacity to identify needs before they turn into exclusion or neglect. Defending community mental health, therefore, also means recognizing and investing in the country’s widespread human capital: in professional expertise, family networks, civic engagement, and a culture of solidarity that has historically positioned Italy as an international reference model of non-institutional, person-centered care grounded in human dignity (WHO, 2022).

From an international perspective, the Italian experience offers a distinctive contrast with healthcare systems such as those of the United Kingdom and the United States, which are currently facing similar pressures related to workforce shortages, increasing demand, and systemic strain. However, unlike more hospital-centered or insurance-driven models, the Italian community-based approach highlights the potential resilience of care systems grounded in continuity, relational proximity, and a strong human-rights framework. These findings suggest that maintaining trust in mental health services under conditions of structural stress may depend not only on resource availability, but also on the underlying cultural and organizational model of care (59).

Given the exploratory and observational nature of the study, the interpretations proposed in this section should be considered as hypothesis-generating reflections grounded in the observed data rather than definitive causal explanations.

These results support previous findings that human-rights-based approaches contribute not only to ethical care but also to the perceived organizational well-being of both users and caregivers (42, 51, 53, 60).

Consistently with prior findings, caregivers continued to report moderate resource constraints, with no significant year-on-year change despite a small, non-significant shift toward lower (i.e., less negative) scores in 2025. This pattern mirrors the 2024 survey and supports the view that structural under-resourcing remains a persistent weakness of services. The resilience of caregiver satisfaction amid austerity may reflect the relational strength of Italy’s community psychiatry network, which remains closely connected to families and local communities (61–63).

Nevertheless, persistent concern over limited resources and staff shortages signals an increasingly fragile balance (64, 65). Caregivers’ growing emphasis on the need for nurses, support staff, and administrative personnel suggests that the cumulative effects of workforce attrition are now visible to service users’ families (66–68). Chronic underinvestment poses a serious threat to the sustainability of this model (50, 69, 70).

This shift toward a more healthcare-focused configuration of perceived needs, characterized by increased demand for nurses and reduced emphasis on psychosocial roles, may reflect an adaptive response to systemic fragility. In contexts of prolonged resource constraints, caregivers may prioritize forms of support perceived as more immediate, tangible, and essential for clinical stability. This pattern can be interpreted as a “survival mode” orientation, in which the focus shifts from comprehensive, recovery-oriented care toward the preservation of basic healthcare functions.

Faced with a perceived more hostile climate toward individuals with psychosocial disabilities, the reaction of family members and other caregivers is to remain connected and satisfied with a community-based network, while clinging to a more healthcare-focused and less psychosocially focused model of care. This shift may not be coincidental, as it appears to occur during a period of heightened strain on the welfare system, when all complex social inclusion interventions are rendered difficult if not impossible, and family members therefore seem to cling to the need for at least some form of healthcare intervention (15, 71, 72).

The desire to increase management staff, as reflected in the 2025 survey, appears to reflect an awareness of the organizational difficulties facing the community mental health care system (73–77).

This paradox may suggest that the ethical foundations of the Italian model remain resilient despite systemic strain, where relational and ethical dimensions of care retain their value despite material constraints. This duality underscores that a values-driven care culture can sustain morale temporarily but cannot indefinitely compensate for material deprivation. If the current trajectory of disinvestment continues, the very principles that define Italy’s mental health reform, community care and respect for human rights, may be undermined (49, 78, 79).

One possible interpretation of these findings relates to processes of adaptive recalibration of expectations among caregivers operating within a context of prolonged systemic strain. In conditions of sustained resource constraints, individuals may progressively adjust their expectations regarding what constitutes acceptable or satisfactory care. This mechanism is conceptually related to the notion of “adaptive preferences,” whereby perceptions of service quality may partly reflect adjustment to constrained institutional environments rather than solely objective improvements in service performance. From this perspective, caregivers’ relatively positive evaluations may reflect a complex interaction between the enduring relational strengths of community-based psychiatry and adaptive processes shaped by structural limitations (13, 67).

In contexts of prolonged systemic strain, individuals may progressively recalibrate their expectations in response to constrained resources, leading to relatively higher satisfaction despite objective limitations. From this perspective, caregivers’ positive evaluations may partly reflect an adjustment to what is perceived as realistically attainable, rather than solely the intrinsic resilience of the services. These two interpretations, resilience of community-based care and adaptive recalibration of expectations, should not be viewed as mutually exclusive, but rather as interacting mechanisms that shape perceptions under conditions of structural fragility. Accordingly, the statistical analyzes should be interpreted primarily as descriptive indicators of group-level tendencies rather than as confirmatory tests of causal relationships. Given the study design, the assumption of independence cannot be formally verified, and potential clustering effects at the level of the care recipient or family context cannot be excluded. As such, the results should be considered indicative of broader perceptual trends rather than precise estimates derived from fully independent units. In this perspective, the present findings should be interpreted as hypothesis-generating signals of evolving caregiver perceptions within a changing healthcare landscape, rather than definitive evidence of causal trends (9, 28, 80).

Future studies with larger samples and more analytically focused designs should incorporate effect size estimation, confidence intervals, and additional robustness analyzes to further strengthen the evidence base. These findings should therefore be interpreted as indicative of group-level perceptual trends rather than definitive evidence of structural changes in service performance.

## 5 Limitations

The cross-sectional design of the study prevents causal inferences regarding the observed differences between the two survey waves. In addition, because the dataset did not include a formal caregiver-service user linkage variable, it cannot be completely excluded that more than one caregiver referred to the same service user, although the recruitment procedure was designed to minimize this possibility. Finally, the study was conducted within a specific regional mental health system, which may limit the generalizability of the findings. An additional limitation concerns the comparison of two independent samples rather than a longitudinal follow-up of the same individuals. Although recruitment procedures were consistent across the two time points, unmeasured differences in sample composition or contextual factors may have influenced the observed results. Therefore, the findings should be interpreted as indicative of differences between samples at the population level rather than individual-level change. Additionally, the absence of participant tracking across time points prevents the exclusion of potential overlap between samples, although this is considered unlikely, although it cannot be formally excluded.

The quality and nature of the caregiving relationship represent another essential dimension that future research should take into account. The type of involvement, whether it arises from family bonds (and the specific nature of those bonds), whether caregiving responsibilities are shared among several relatives, or whether the relationship is based on friendship, can influence caregivers’ perceptions. Furthermore, factors such as the care recipient’s level of autonomy and the severity or persistence of their condition may significantly shape how caregivers perceive gaps or shortcomings in available services, as well as the overall dynamics of the relationship. These aspects warrant careful examination in future studies. Furthermore, the study did not collect information linking caregivers to specific service users. Therefore, it was not possible to verify whether multiple respondents referred to the same individual, and potential clustering within the same family or care context cannot be formally excluded.

An additional limitation concerns the difference in centers composition between the two samples, as the 2024 data were collected in four centers, whereas the 2025 sample was drawn from three of these settings. Variations in organizational context, service structure, or local practices across centers may have influenced caregivers’ perceptions and contributed to the observed differences between samples. Therefore, part of the variability observed may reflect site-related factors rather than differences associated with the temporal context alone.

A limitation concerns the lack of systematic recording of non-participation. The study did not collect data on the number of eligible caregivers who declined to participate, nor on their characteristics. Consequently, it is not possible to assess the extent of potential non-response bias or to determine whether participants differ systematically from non-participants. This limitation is inherent to the naturalistic recruitment approach adopted and should be considered when interpreting the findings.

Moreover, the absence of detailed clinical and caregiving-related variables, such as the severity of the care recipient’s condition, duration of caregiving, and level of involvement, limits the ability to fully assess comparability between the two samples. These unmeasured factors may have influenced caregivers’ perceptions and should be considered in interpreting the results.

A key methodological limitation concerns the assumption of independence between observations. The study design did not allow verification of whether multiple caregivers referred to the same care recipient or whether any individuals may have participated in both survey waves. As a result, potential clustering effects at the level of the care recipient or family context cannot be excluded, and the assumption of independence underlying the statistical analyzes may be partially violated.

A major limitation of this study is the use of convenience sampling in routine clinical settings, which may have introduced selection bias. Caregivers who agreed to participate may differ systematically from those who declined, potentially influencing the observed patterns of responses. In addition, the absence of a formal record of non-participation prevents a direct assessment of non-response bias and limits the ability to evaluate the representativeness of the sample. This limitation should be considered when interpreting the findings, particularly in light of current international standards promoting gender-inclusive research practices.

The absence of linkage between caregivers and care recipients prevents the application of clustered or multilevel analytical approaches. Future studies should address this limitation by incorporating designs that allow the identification of caregiving units while preserving participant confidentiality.

The use of parametric tests on Likert-type data may be sensitive to deviations from normality, including skewed distributions. Although ANOVA is generally considered robust under these conditions, the potential influence of non-normality cannot be entirely excluded.

## 6 Conclusion

Informal caregivers in the two samples examined expressed generally high levels of satisfaction with mental health services and confidence in their respect for human rights. These findings suggest that positive perceptions of the Italian community-based psychiatric model remain evident across the two samples. However, caregivers’ growing concern about staff shortages and diminishing resources points to a critical juncture. Sustaining the Italian model requires not only preserving its ethical foundations but also ensuring adequate investment in human and structural resources. Strengthening multidisciplinary teams and reaffirming the human-rights orientation of care are essential steps to protect the future of community psychiatry in Italy.

These results should be interpreted as reflecting differences between two independent samples rather than evidence of true temporal change, and therefore provide exploratory insight into caregiver perceptions under conditions of systemic strain.

## 7 Policy implications

### 7.1 Reinvest in community mental health services

Adequate staffing and infrastructure are essential to sustain Italy’s community-based care model.

### 7.2 Protect and expand law 180 principles

Policymakers should reaffirm the commitment to deinstitutionalization and community integration.

### 7.3 Adopt human-rights-based quality frameworks

The WHO *QualityRights* program should be systematically implemented across all health sectors.

### 7.4 Monitor caregiver perspectives

Periodic surveys using the WWRR can serve as an early-warning system for detecting organizational distress.

### 7.5 Integrate caregivers into service planning

Their perceptions provide critical insights into service effectiveness, ethical climate, and social sustainability.

## Data Availability

The original contributions presented in the study are included in the article/supplementary material. Further inquiries can be directed to the corresponding author.
